# Combined low initial DNA damage and high radiation-induced apoptosis confers clinical resistance to long-term toxicity in breast cancer patients treated with high-dose radiotherapy

**DOI:** 10.1186/1748-717X-6-60

**Published:** 2011-06-06

**Authors:** Luis Alberto Henríquez-Hernández, Ruth Carmona-Vigo, Beatriz Pinar, Elisa Bordón, Marta Lloret, María Isabel Núñez, Carlos Rodríguez-Gallego, Pedro C Lara

**Affiliations:** 1 Radiation Oncology Department, Hospital Universitario de Gran Canaria Dr. Negrín, Spain; 2 Instituto Canario de Investigación del Cáncer (ICIC), Spain; 3 Clinical Sciences Department, Universidad de Las Palmas de Gran Canaria, Spain; 4 Radiology Department, Hospital Universitario San Cecilio, Granada, Spain; 5 Immunology Department, Hospital Universitario de Gran Canaria Dr. Negrín, Spain

## Abstract

**Background:**

Either higher levels of initial DNA damage or lower levels of radiation-induced apoptosis in peripheral blood lymphocytes have been associated to increased risk for develop late radiation-induced toxicity. It has been recently published that these two predictive tests are inversely related. The aim of the present study was to investigate the combined role of both tests in relation to clinical radiation-induced toxicity in a set of breast cancer patients treated with high dose hyperfractionated radical radiotherapy.

**Methods:**

Peripheral blood lymphocytes were taken from 26 consecutive patients with locally advanced breast carcinoma treated with high-dose hyperfractioned radical radiotherapy. Acute and late cutaneous and subcutaneous toxicity was evaluated using the Radiation Therapy Oncology Group morbidity scoring schema. The mean follow-up of survivors (n = 13) was 197.23 months. Radiosensitivity of lymphocytes was quantified as the initial number of DNA double-strand breaks induced per Gy and per DNA unit (200 Mbp). Radiation-induced apoptosis (RIA) at 1, 2 and 8 Gy was measured by flow cytometry using annexin V/propidium iodide.

**Results:**

Mean DSB/Gy/DNA unit obtained was 1.70 ± 0.83 (range 0.63-4.08; median, 1.46). Radiation-induced apoptosis increased with radiation dose (median 12.36, 17.79 and 24.83 for 1, 2, and 8 Gy respectively). We observed that those "expected resistant patients" (DSB values lower than 1.78 DSB/Gy per 200 Mbp and RIA values over 9.58, 14.40 or 24.83 for 1, 2 and 8 Gy respectively) were at low risk of suffer severe subcutaneous late toxicity (HR 0.223, 95%CI 0.073-0.678, *P *= 0.008; HR 0.206, 95%CI 0.063-0.677, *P *= 0.009; HR 0.239, 95%CI 0.062-0.929, *P *= 0.039, for RIA at 1, 2 and 8 Gy respectively) in multivariate analysis.

**Conclusions:**

A radiation-resistant profile is proposed, where those patients who presented lower levels of initial DNA damage and higher levels of radiation induced apoptosis were at low risk of suffer severe subcutaneous late toxicity after clinical treatment at high radiation doses in our series. However, due to the small sample size, other prospective studies with higher number of patients are needed to validate these results.

## Background

Locally advanced breast cancer (LABC) is a relatively infrequently tumour which poses a significant clinical challenge. The management of LABC has evolved considerably. Initially, patients with LABC were treated with radical mastectomy [1,2]; thereafter, systemic therapy was subsequently incorporated along with surgery and radiotherapy (RT) [3]. However, even with such combined modality therapy, the long-term survival rate is approximately 50% among patients with LABC [4]. In cases with inadequate response to neoadjuvant systemic therapies and inability to perform surgery, RT is the only possible treatment [5].

Better local control outcomes, with acceptable toxicity, have been obtained by using high total doses of radiation administered in two small fractions per day (hyperfractionation, HF) [6]. HF allows escalation of the biologically effective dose to the tumour without a significant increase in late complications [7]. The radio therapeutic doses received by the patient are limited by the tolerance of the normal tissues. Different patients given a standardized treatment can exhibit a range of normal acute and/or late tissue reactions [8,9]. Thus, there is both a dose dependence and a variability in individual radiosensitivity, where genetic [10,11] and constitutional factors [9,12] inherit to each patient could exert an influence.

The prediction of radiation-induced toxicity could help to select the most appropriate treatment for each patient. Many predictive factors have been described, including initial DNA damage [13], cell apoptosis [14], or gene expression patterns [15,16]. In previous studies, we have reported an association between the initial number of DNA double-strand breaks (DSB) induced by x-rays in peripheral blood lymphocytes (PBL) and radiation-toxicity [17,18]. Thus, increasing numbers of radiation induced DSB were related to severe late subcutaneous toxicity in LABC patients treated with HF [18]. In the other hand, determination of radiation-induced apoptosis (RIA) in PBL by flow cytometry analysis has also been proposed as an approach for predicting normal tissue responses following radiotherapy [19,20]. Patients suffering of late toxicity after RT showed reduced rates of RIA in several tumour locations [20-22]. Moreover, we have recently reported an inverse association between the initial DNA damage and RIA in LABC patients [23].

Taking into account the above background and our previously observations, we explored the clinical association between initial DNA damage and RIA in relation to radiation-induced toxicity in the set of LABC patients treated with high dose HF radical RT with long-term follow-up where this association have been previously observed [23].

## Methods

### Characteristics of Patients

Twenty-six consecutive patients diagnosed in our institution with locally advanced/inflammatory breast cancer were recruited prospectively for the study after they signed informed consent to their participation. The study was approved by the Research and Ethics Committee of our Institution. All patients were treated between 1992 and 1997; blood samples for radiosensitivity testing were extracted between February and December 1998. All the analyses were double-blinded to ensure their reliability. Mean age of patients was 57.62 ± 12.9 years (range 30-83). The majority of patients were postmenopausal (69.2%), presented bra size over 100 (65.4%), and non-inflammatory LABC (73.1%). Characteristics of patients are detailed in Table 1. Evaluation of clinical toxicity was made in each visit. The Radiotherapy Oncology Group (RTOG) morbidity score system was used to classify the toxicity of patients. Acute toxicity was evaluated during and at the end of RT. Late cutaneous and subcutaneous toxicity was evaluated every three months during the first two years, every six months to five years, and thereafter annually. At the end of the analysis (January 2011), the mean clinical follow-up of survivors (n = 13) was 197.23 months (range 155-228). The time point finally used for analysis corresponds to the last evaluation. Clinical toxicities of patients are detailed in Table 2.

**Table 1:** Characteristics of patients studied

	N (%)	Mean ± SD	Median (Range)
Age		57.62 **± **12.9	60 (30-83)
<60 years	12 (46.2)		
≥60 years	14 (53.8)		
Menopause			
Premenopausal	8 (30.8)		
Postmenopausal	18 (69.2)		
Tumor type			
Inflammatory	7 (26.9)		
Non-inflammatory	19 (73.1)		
Tumor size			
T3	1 (3.8)		
T4a-T4b	18 (69.2)		
T4c-T4d	7 (27.0)		
Nodes			
N0	18 (69.2)		
N1-N2	8 (30.8)		
Metastasis			
M0	24 (92.3)		
M1	2 (7.7)		
Bra size		100 ± 10.6	100 (80-120)
<100	9 (34.6)		
≥100	17 (65.4)		
Systemic treatment			
Chemotherapy	4 (15.4)		
Hormonal therapy	5 (19.2)		
Chemotherapy-hormonal therapy	17 (65.4)		
Received dose (Gy)		78.48 **± **5.7	81.60 (64.8-81.6)
<81.6	7 (26.9)		
≥81.6	19 (73.1)		
Maximum dose (Gy)		87.36 **± **8.8	89.76 (62.8-101.7)
<89.8	15 (57.7)		
≥89.8	11 (42.3)		

**Table 2:** Number of patients who developed acute/late toxicity due to radiotherapy

	Acute Toxicity	Late Toxicity	
	
Grade	*Cutaneous*	*Cutaneous*	*Subcutaneous*
1	6 (23.1)	0 (0.0)	1 (3.8)
2	12 (46.2)	16 (61.5)	5 (19.3)
3	8 (30.8)	10 (38.5)	19 (73.1)
4	0 (0.0)	0 (0.0)	1 (3.8)

Numbers in brackets represent the percentage.

### Radiation Treatment

Patients were treated with a dose-escalation radiation therapy schedule using hyperfractionation. All patients received 60 Gy to the whole breast over a period of 5 weeks in two daily fractions of 1.2 Gy, separated by at least 6 h on 5 days each week. A boost covering the tumour plus margins was prescribed at a dose of 9.4-21.6 Gy [17]. Peripheral nodes were treated by conventional fractionation (1.8/2Gy/day) at doses of 50-70 Gy. Supraclavicular and axillary lymph node areas were treated with an anterior field and a posterior axillary compensating field. Doses were prescribed to the mid-plane of the axilla and at a depth of 3 cm in the supraclavicular area. The internal mammary chain was treated by a direct anterior field with the dose prescribed at depth of 3 cm. Doses to the breast ranged from 64.8 Gy to 81.6 Gy (mean 77.5 ± 5.7 Gy; median 81.6 Gy). Maximum point doses ranged from 62.8 to 101.7 Gy (mean 87.4 ± 8.8; median 89.7 Gy).

### Analysis of Initial DNA Damage

Data related to initial DNA damage were obtained from our files [17]. Shortly, mononuclear cells were isolated from blood of patients, resuspended in cold DMEM, and mixed with 1% ultra-low-melting-point agarose to obtain 250 μl plugs. Irradiation on ice was performed using a ^60^Co source (rate dose 1.5 Gy/min, approximately) as previously reported [17]. Plugs were held 1 hour at 4°C and incubated at 37°C for 24 hours. Initial radiation-induced DNA damage in PBL was measured by pulsed-field gel electrophoresis (PFGE) as previously described [24], and data are summarized in Table 3.

**Table 3:** Apoptosis data obtained after the irradiation of PBL at 1, 2 and 8 Gy

	Mean ± SD	Median (range)	Tertiles	*P*
DSB/Gy/DNA unit	1.70 ± 0.83	1.46 (0.63-4.08)	1.28-1.78	0.290
RIA 1Gy	13.33 ± 7.26	12.36 (2.51-29.00)	9.58-15.52	0.971
RIA 2Gy	18.20 ± 7.82	17.79 (4.17-32.08)	14.40-22.43	0.996
RIA 8Gy	29.70 ± 10.05	30.44 (9.02-44.10)	24.83-34.40	0.977
α	13.08 ± 7.21	12.64 (1.64-26.63)	9.91-15.63	0.994
β	7.93 ± 2.68	7.85 (3.18-12.57)	7.14-9.29	0.943

*Abbreviations: *DSB/Gy/DNA unit = double-strand breaks induced per Gy and per 200 Mbp; RIA = radiation-induced apoptosis at 1, 2 and 8 Gy after 24 hours. α and β are the constants that define the model. *P *values were obtained after a Kolmogorov-Smirnov test.

### Apoptosis assay and flow cytometry

RIA analyses were performed as previously reported [21,22]. PBL were irradiated with 0, 1, 2 and 8 Gy. After irradiation, samples were incubated for 24 hours at 37°C and 5% CO_2_. After extraction of cellular pellet, it was resuspended in 100 μl Annexin V buffer Kit (Pharmingen, Becton Dickinson). After the addition of 4 μl of Annexin-V-FITC and 10 μl of propidium iodure (PI), cells were incubated during 15 minutes at room temperature in the dark. Finally, 400 μl of Annexin V buffer Kit were added. Every assay was made in triplicate.

The flow cytometry analysis was performed in a FACScalibur (Becton Dickinson, San José, CA) using a 488 nm argon laser, and each sample was analyzed in a Macintosh Quadra 650 minicomputer (Apple computer Inc., Cupertino, CA) as previously reported [25]. Data were analyzed using the CellQuest program (Becton Dickinson, San José, CA) calculating early and late apoptosis levels. RIA is defined as the percentage of total PBL death induced by the radiation dose minus the spontaneous cell death (control, 0 Gy).

### Statistical analyses

Statistical analyses were performed using the SPSS Statistical Package (version 15.0 for Windows). The cut-off values for continuous variables were the median and the tertiles of the distribution, as previously reported [17,23]. Univariate and multivariate analyses were performed using Cox regression. All tests were two sided and statistical significance level was established for a *P *value less than 0.05. All samples were processed anonymously.

## Results and Discussion

### Radiation-induced toxicity in breast cancer patients

The actuarial probability of being free of severe late cutaneous toxicity, nine-teen years after radiation therapy, was 61.5%, while only 19.2% were free of severe late subcutaneous toxicity. In a previous observation, 10 years after RT [17], 65% of patients were free of severe late cutaneous toxicity (χ^2 ^test, *P *= 0.463); while 29% were free of severe late subcutaneous toxicity (χ^2 ^test, *P *= 0.031). Severe subcutaneous toxicity is related to breast shrinkage, fibrosis and sometimes pain. Late radiation-induced reaction occurs after a latency period of >90 days (typical range 0.5-5 years). The latency period in animals is known to be shorter after higher doses, and in humans, it is even >5 years for moderate doses or for very late reacting tissues. Late damage progresses over time, and it is important to highlight that doses believed safe at 5 years may result in serious late side effects beyond the 5-year period with any treatment protocol [26]. For this, the ability to predict late effects in the treated breast is of great importance, especially when an unconventional treatment schedule is prescribed. In univariate analysis (simple Cox regression), severe subcutaneous late toxicity (grades 3-4) was related to bra size-estimated breast volume (*P *= 0.037) (Table 4). Breast size is strongly related to late changes in breast appearance possible because greater radiation changes are related to greater dose inhomogeneity in women with large breasts [12,17,27].

**Table 4:** Distribution of patients according to expected radiation sensitivity after the irradiation of peripheral blood lymphocytes at 1, 2 and 8 Gy

Expected radiation sensitivity	RIA 1 Gy	RIA 2 Gy	RIA 8 Gy
High (↑ DSB, ↓ RIA)	2	1	3
Intermediate*	13	15	10
Low (↓ DSB, ↑ RIA)	11	10	13
	26	26	26

*Abbreviations: *DSB = DNA double-strand breaks; RIA = radiation-induced apoptosis

*Intermediate: patients showing ↑ DSB, ↑ RIA; or ↓ DSB, ↓ RIA.

### Initial DNA damage levels in breast cancer patients

Initial DNA damage was determined as radiation-induced double-strand breaks (DSB) in irradiated lymphocyte from all 26 LABC patients. There was a wide variation in DSB among patients (Table 3) with a mean value of 1.70 ± 0.83 DSB/Gy per 200 Mbp (median, 1.46; range, 0.63-4.08). These results support the suggestion that variation in cell radiosensitivity can be detected *in vitro *using radiosensitivity assays on lymphocytes derived from normal tissues of cancer patients prior to radiotherapy [18,28-30]. This wide variation in DNA DSB can be attributed to variation between individuals more than to variation due to technical or sampling errors [18,31,32]. Initial DNA damage followed a normal distribution (Kolmogorov-Smirnov test, *P *> 0.05), and data obtained from the present group of patients matched previously published results for breast cancer patients [17,18]. However, other molecular events such as DNA repair foci or DNA-loops should be taken into account for the correct interpretation of data. It has been observed that DNA DSB in residual foci and relaxation of DNA-loops may be linked to induction of radiation-induced apoptosis in lymphocytes [33-35].

We have previously demonstrated a relation between the sensitivity of *in vitro*-irradiated peripheral blood lymphocytes and the risk of developing late toxic effects after RT in the present set of patients [17]. However, the predictive value of initial DNA damage is controversial and different findings have been reported on this regard. Thus, we agree with some authors [28,30,36] and we disagree with some others [37]. Moreover, more initial DSB have been detected in lymphocytes from normal patients as compared to radiosensitive [38]. In our opinion, it is important to highlight that the predictive role of initial DNA damage was observed in patients treated with high-dose of radiation, where the toxicity reactions are more evident. Differences in the protocol treatment (RT schedule: dose and type of fractionation) and in the methodology used (PFGE, comet assay, gamma-H2AX induction) could help to explain the discrepancies observed.

### Radiation-induced apoptosis in breast cancer patients

Data of RIA were available in all 26 breast cancer patients as shown in Table 3. RIA increased with radiation dose and data fitted to a semi logarithmic model as follows: RIA = β ln(Gy) + α. This mathematical model was defined by two constants: the coefficient in origin α (determining the spontaneous apoptosis) and the coefficient β (defining the slope of the curve) [21,22,25,39]. As expected, RIA at 1, 2 and 8 Gy, as well as α and β constants followed a normal distribution (Kolmogorov-Smirnov test, *P *> 0.05). There is an important variation in the *ex vivo *susceptibility of normal cells against ionizing radiation. It has been suggested that the radiation-induced damage measured on lymphocytes could be proportional to the acute damage evaluated on the skin of treated patients [40]. Anyhow, it is possible to estimate the cellular radiosensitivity of PBL of patients analyzing the RIA rate by annexin V/PI staining flow cytometric analysis, defining an intrinsic individual value of radiosensitivity inherit to each patient.

Radiation-induced apoptosis has been proposed as a reliable method for prediction of normal tissue toxicity after radiotherapy by us [21,22] and other authors [14,19,20]. However, some other studies reported no correlations between individual radiosensitivity of cancer patients and radiation-induced apoptosis in PBLs [41,42]. The lack of uniformity in experimental design helps to understand these differences. Thus, the cells used in the assay (total PBL, Epstein-Barr virus-transformed lymphoblastoid cell lines, CD(3+) lymphocytes), the radiation protocol, or the analysis strategy are critical to make possible the comparison among studies.

### Association of initial DNA damage and radiation-induced apoptosis with normal tissue toxicity

As previously published, increasing numbers of radiation induced DSB were related to severe late toxicity in breast cancer patients [17]. Thus, among patients receiving the highest radiation doses (81.6 Gy), those who showed higher levels of initial DNA damage had a greater risk of severe subcutaneous toxicity. In the present set of patients, no association was observed between DNA DSB or RIA (at any radiation dose), α or β constants and normal tissue toxicity, possibly due to the small sample size (data not shown). An association between the initial DNA damage and the radiation-induced apoptosis, as a consequence of x-ray, may exist [43,44]. DNA DSB are assumed to be the most important lesion to induce apoptosis [45]. Depending on the severity of the DNA damage and the cell type involved, cells may undergo apoptosis instead of attempting to repair the damage [46]. Lymphocytes are particularly sensitive to apoptosis, partly because they induce Bax expression in response to ionizing radiation exposure [46]. Lymphocytes from patients who suffered Ataxia-telangiectasia, Bloom syndrome, or Fanconi anaemia showed absence of induction of p53 and lower levels of Bax [47-49]. Apoptosis is initiated following DSB through an ATM-directed pathway [50]. This could explain the fact that patients affected by the Ataxia-Telangiectasia syndrome show the lowest rates of RIA. In that sense, we have recently reported an inverse association between the initial DNA damage and RIA in LABC patients [23]. Defective apoptotic response to radiation in PBLs could help to explain this inverse relation [14].

According to the above observations, high initial DNA damage [17] or low radiation-induced apoptosis [14,20-22,25,51] would confer sensitivity to long-term toxicity, separately. In the present study, we tried to disclose the predictive value of both parameters in a combined form. The percentage of patients developing severe late toxicity determines the maximum acceptable radiation dose. Generally, an adverse effect frequency of 5%-10% is considered acceptable [52]. We observed that 7.6% (range 3.8-11.5%) of our patients suffered from severe complications (2, 1, and 3 out of 26 patients analyzed at 1, 2 and 8 Gy respectively) (Table 4). Because this subset of patients is too small, we focused on the expected most resistant patients to RT: those who presented low initial DNA damage and high radiation-induced apoptosis (Table 4). Thus, we considered "resistant patients" those who presented DSB values lower than 1.78 DSB/Gy per 200 Mbp (two lower thirds of the distribution) and RIA values over 9.58, 14.40 or 24.83 for 1, 2 and 8 Gy respectively (two upper thirds of the distribution) (Table 3). We did not observe any association with late toxicity in the whole series, in univariate analysis. However, order to the higher received dose (≥81.6 Gy), we observed that severe subcutaneous late toxicity (grades 3-4) was related to this radiation-resistance profile in patients treated with higher dose of radiation (simple Cox regression, Table 5). Those patients treated at very high doses (≥81.6 Gy) and who presented this radiation-resistance pattern were at low risk of suffer severe subcutaneous late toxicity (Table 5). Furthermore, in multivariate analysis in the whole series, severe subcutaneous late toxicity was related to the received dose (HR 1.138, 95%CI 1.003-1.291, *P *= 0.045), the bra size-estimated volume (HR 1.073, 95%CI 1.004-1.147, *P *= 0.038), and with this radiation-resistant profile (HR 0.223, 95%CI 0.073-0.678, *P *= 0.008; HR 0.206, 95%CI 0.063-0.677, *P *= 0.009; HR 0.239, 95%CI 0.062-0.929, *P *= 0.039, for RIA at 1, 2 and 8 Gy, respectively) (Table 6). Thus, those patients who presented lower levels of initial DNA damage and higher levels of radiation induced apoptosis were at low risk of suffer severe subcutaneous late toxicity. No relation was found with acute or late cutaneous toxicity. The close relation between chromosome fragment production and killing in many cell systems has been important in linking DNA DSB to death, because it is a natural step to relate DNA strand breakage to chromosome breakage. However, the recognition that apoptosis may be an important mode of radiation-induced death in some cell types raise the possibility that other types of damage may induce apoptosis [13]. A significant association was observed for the first time between these variables, both considered as predictive factors for radiation toxicity, and normal tissue damage.

**Table 5:** Univariate analysis for grades 3-4 late subcutaneous toxicity in the whole series of patients (n = 26) and in patients who received higher doses of RT (n = 19)

	HR	(95% CI)	*P*
**Whole series**			
Age	1.012	(0.975-1.012)	0.535
Received dose	1.079	(0.980-1.189)	0.123
Maximum dose	1.054	(0.991-1.121)	0.096
Bra size	1.056	(1.003-1.111)	0.037
Systemic treatment	1.084	(0.351-3.347)	0.888
Low DSB-High RIA 1Gy	0.564	(0.233-1.370)	0.206
Low DSB-High RIA 2Gy	0.510	(0.204-1.277)	0.150
Low DSB-High RIA 8Gy	0.642	(0.270-1.523)	0.314
**Higher dose (≥81.6Gy)**			
Low DSB-High RIA 1Gy	0.252	(0.077-0.826)	0.023
Low DSB-High RIA 2Gy	0.197	(0.053-0.735)	0.016
Low DSB-High RIA 8Gy	0.240	(0.074-0.778)	0.017

*Abbreviations: *HR = hazard ratio; CI = confidence interval

**Table 6:** Multivariate analysis for grades 3-4 late subcutaneous toxicity in the whole series of patients (n = 26)

	HR	(95% CI)	*P*
**Whole series**			
Age	1.044	(0.986-1.106)	0.139
Received dose	1.138	(1.003-1.291)	0.045
Bra size	1.073	(1.004-1.147)	0.038
Systemic treatment	1.155	(0.199-6.697)	0.873
Low DSB-High RIA 1Gy	0.223	(0.073-0.678)	0.008
Low DSB-High RIA 2Gy	0.206	(0.063-0.677)	0.009
Low DSB-High RIA 8Gy	0.239	(0.062-0.929)	0.039

*Abbreviations: *HR = hazard ratio; CI = confidence interval

## Conclusions

Initial DNA double-strand breaks and radiation-induced apoptosis in peripheral blood lymphocytes have been proposed as reliable methods for prediction of radiation-induced late toxicity in normal tissues [11,17,20]. We have observed, for the first time, a combined role of both parameters. Thus, we propose a radiation-resistance profile where those patients who present lower levels of initial DNA damage and higher levels of radiation induced apoptosis were at low risk of suffer severe subcutaneous late toxicity in our series. This finding opens the possibility to develop new predictor assays taking into account the initial DNA damage and radiation-induced apoptosis levels, and introduces new data which may help to understand and define the complex mechanisms behind the normal tissue toxicity. Nonetheless, due to the small sample size, the present results need to be validated in bigger clinical series.

## List of abbreviations

DSB: double-strand Break; HF: hyperfractionation; HR: hazard ratio; CI: confidence interval; LABC: locally advanced breast cancer; PBL: peripheral blood lymphocytes; PI: propidium iodide; RIA: radiation-induced Apoptosis; RT: radiotherapy.

## Competing interests

The authors report no conflicts of interest. The authors alone are responsible for the content and writing of the paper.

## Authors' contributions

LAHH has written the manuscript, has participated in the statistical analysis, has made tables and has been involved in type of packaging likewise in the submission process. RCV has made the last revision of patients as well as the update of the medical records. BP and ML have made the selection of patients, the evaluation of clinical variables and grade of toxicity as well as all the aspects related with the patients selected, including the treatment. EB and CRG have made the cell experiments with lymphocytes, irradiation of cells, flow cytometry experiments and data acquisition. MIN has been involved in conception and design of the study and has made the DNA-DSB experiments and analyses. PCL has been involved in conception and design of the study and in drafting the manuscript and has given final approval of the version to be published. All authors read and approved the final manuscript.
